# DUQuE quality management measures: associations between quality management at hospital and pathway levels

**DOI:** 10.1093/intqhc/mzu020

**Published:** 2014-03-09

**Authors:** Cordula Wagner, Oliver Groene, Caroline A. Thompson, Maral Dersarkissian, Niek S. Klazinga, Onyebuchi A. Arah, Rosa Suñol, on behalf of the DUQuE Project Consortium, N Klazinga, DS Kringos, K Lombarts, T Plochg, MA Lopez, M Secanell, R Sunol, P Vallejo, P Bartels, S Kristensen, P Michel, F Saillour-Glenisson, F Vlcek, M Car, S Jones, E Klaus, P Garel, K Hanslik, M Saluvan, C Bruneau, A Depaigne-Loth, C Shaw, A Hammer, O Ommen, H Pfaff, O Groene, D Botje, C Wagner, H Kutaj-Wasikowska, B Kutryba, A Escoval, M Franca, F Almeman, H Kus, K Ozturk, R Mannion, OA Arah, A Chow, M DerSarkissian, C Thompson, A Wang, A Thompson

**Affiliations:** 1 NIVEL, Netherlands Institute for Health Services Research, Utrecht, The Netherlands; 2 Department of Public and Occupational Health, EMGO+ Institute for Health and Care Research, VU University Medical Center, Amsterdam, The Netherlands; 3 Department of Health Services Research and Policy, London School of Hygiene & Tropical Medicine, London, UK; 4 Department of Epidemiology, Fielding School of Public Health, University of California, Los Angeles (UCLA), Los Angeles, CA, USA; 5 Palo Alto Medical Foundation Research Institute, Palo Alto, CA, USA; 6 Department of Public Health, Academic Medical Center, University of Amsterdam, Amsterdam, The Netherlands; 7 UCLA Center for Health Policy Research, Los Angeles, CA, USA; 8 Avedis Donabedian Research Institute (FAD), Universitat Autonoma de Barcelona, Barcelona, Spain; 9 Red de Investigación en Servicios de Salud en Enfermedades Crónicas (REDISSEC), Spain

**Keywords:** quality management, quality improvement, external quality assessment, measurement of quality, organization science, healthcare system, patient safety, hospital care

## Abstract

**Objective:**

The assessment of integral quality management (QM) in a hospital requires measurement and monitoring from different perspectives and at various levels of care delivery. Within the DUQuE project (Deepening our Understanding of Quality improvement in Europe), seven measures for QM were developed. This study investigates the relationships between the various quality measures.

**Design:**

It is a multi-level, cross-sectional, mixed-method study.

**Setting and Participants:**

As part of the DUQuE project, we invited a random sample of 74 hospitals in 7 countries. The quality managers of these hospitals were the main respondents. Furthermore, data of site visits of external surveyors assessing the participating hospitals were used.

**Main Outcome Measures:**

Three measures of QM at hospitals level focusing on integral systems (QMSI), compliance with the Plan-Do-Study-Act quality improvement cycle (QMCI) and implementation of clinical quality (CQII). Four measures of QM activities at care pathway level focusing on Specialized expertise and responsibility (SER), Evidence-based organization of pathways (EBOP), Patient safety strategies (PSS) and Clinical review (CR).

**Results:**

Positive significant associations were found between the three hospitals level QM measures. Results of the relationships between levels were mixed and showed most associations between QMCI and department-level QM measures for all four types of departments. QMSI was associated with PSS in all types of departments.

**Conclusion:**

By using the seven measures of QM, it is possible to get a more comprehensive picture of the maturity of QM in hospitals, with regard to the different levels and across various types of hospital departments.

## Introduction

Hospitals are complex, hierarchical, multi-level organizations. To assure and continuously improve the quality and safety of healthcare delivery and patient outcomes, it is important to have quality strategies in place at every level [1]. Quality strategies are tools, procedures or activities aimed at improving patient care. Types of strategies and the extent of their implementation can be different for the various levels or departments in a hospital. In order to improve patient care, it is not enough to have a quality management (QM) system at hospital level only [2, 3]. Patient-related outcomes depend on the direct activities of professionals at the sharp end of care processes [4]. In the DUQuE project ‘Deepening our Understanding of Quality Improvement in Europe’, we conceptualized QM as a systematic process of identifying, assessing and taking action to maintain and improve the quality of care (structures, processes and outcomes) throughout the hospitals [5]. This definition implies that the evaluation of the development and implementation of QM for a whole hospital asks for detailed measurement and monitoring of QM from different perspectives and at various levels of care delivery. A review of instruments to assess the implementation of QM systems showed that the various developed instruments assess different aspects of QM, but none of the instruments cover the various levels within a hospital. Besides differences, there are also common domains in existing instruments focusing on process management, human resources, leadership, monitoring based on indicators, structures and responsibilities, and patient involvement [6]. These domains are a combination of the managerial aspects of a QM systems and more contextual factors.

Within the DUQuE project, we therefore developed and tested three indices at hospital level and four scales at pathway level (Box 1). A scale represents multiple items measuring a single construct or dimension, and an Index summarizes items or scales representing multiple dimensions [7]. By using these seven measures, it is possible to get a more comprehensive picture of the implementation of QM in hospitals, with regard to the different levels and across various hospital departments. In earlier articles, the psychometric properties of the QM indices and scales have been determined [8–10]. Therefore, the aim of this study was to investigate the relationships among the various measures of hospital-level QM indices and pathway-level QM scales.

Box 1 **DUQuE quality management indices and scales**

Quality Management indices at hospital level

 Quality Management System Index (QMSI)

 Quality Management Compliance Index (QMCI)

 Clinical Quality Implementation Index (CQII)

Quality Management Scales at pathway level

 Specialized expertise and responsibility (SER)

 Evidence-based organization of pathways (EBOP)

 Patient safety strategies (PSS)

 Clinical review (CR)

Two questions will be addressed: (1) is the more holistic quality management system (QMSI) associated with the compliance and use of the quality improvement cycle (QMCI) and the implementation of clinical quality activities (CQII) at hospital level? and (2) to what extent are hospital QM strategies related to QM activities at pathway level?

### Theoretical considerations

**Figure 1:**
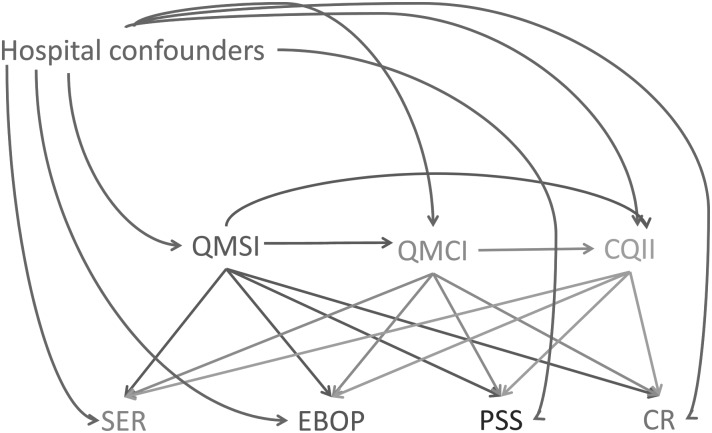
Directed acyclic graph showing the relationships between study variables.

Quality management as a managerial function and responsibility of the hospital board has to be embedded into the organizational infrastructure [11, 12]. As such QM is assumed to support effective care pathways and positive patient outcomes. We assumed that the success of QM depends on whether there is a QM system (QMSI) in place, the extent of compliance with the quality improvement cycle Plan-Do-Study-Act (QMCI), the focus of professionals on clinical outcomes (CQII) at hospital level and the implementation of QM activities at pathway level. Because of the complexity of hospitals and the various professional groups at pathway level, there is not a direct and linear relation between decisions by the board of the hospital and patient care and outcomes. Therefore, it is important to establish whether there is a relationship between hospital- and pathway-level QM first. Furthermore, it is important to investigate the horizontal relation between the three QM indices. The three measures differ from each other in their scope (from broad and generic to smaller and clinical focused) and in the responsibility (managers versus clinician). Figure 1 shows the expected relations between the seven QM measures. In Box 1, the full names of the measures are given.

We assumed that the development of a good infrastructure of a QMS is positively related to the compliance of QM activities and the implementation of clinical QM activities at hospital level (QMSI → QMCI → CQII). Strong QM at hospital level is positively related to activities at pathway level by stimulating specialized expertise and assigning responsibilities, by organizing pathways with regard to existing evidence, by developing safety strategies, and by organizing clinical reviews. Each of the three hospital-level measures is expected to have a positive relation with the four pathway-level measures. The analysis will show whether all seven measures will give distinct information and follow the expected direction of relationship.

## Methods

### Setting and participants

We used survey and audit data on QM activities in a sample of 74 acute care hospitals in 7 European countries, e.g. Czech Republic, France, Germany, Poland, Portugal, Spain and Turkey. The online questionnaire measuring QMSI were completed by the quality managers of the hospitals. Respondents who did not respond within 4 weeks were sent a reminder. The other indices and scales (Box 1) were measured using structured questionnaires filled in by an external auditor during a one-day site-visit of the hospital and interviews with various managers and professionals of the hospital. Ethical approval was gained by the project coordinator at the Bioethics Committee of the Health Department of the Government of Catalonia (Spain) and each country complied with the confidentiality issues according to national legislation or standards of practice available in each country [13].

### Measures used

#### QMSI

The QMSI was developed based on previous research by the research team and following a systematic review of the literature on conceptual models and measurement instruments [1, 3, 6]. The quality managers were asked to answer questions divided over several dimensions. Psychometric analysis showed that the QMSI is a reliable instrument consisting of nine dimensions: quality policy documents (three items), quality monitoring by the board (five items), training of professionals (nine items), formal protocols for infection control (five items), formal protocols for medication and patient handling (four items), analyzing performance of care processes (eight items), analyzing performance of care professionals (three items), analyzing feedback patient experiences (three items) and evaluate results (six items). On each item, the respondent could answer on a four-point-Likert-type scale (range 0–3), with answer categories ranging from ‘Not available to Fully implemented’ and from ‘Disagree to Agree’. The maturity of the management system is expressed as an index (0–27), based on the extent of implementation of QI activities. In other words: the maturity index tells us something about how mature/well implemented the activity is within the hospital: does it exist? Is it partly implemented? Fully implemented in one department? Fully implemented in all departments? [8].

#### QMCI

The Quality Management Compliance Index (QMCI) consists of four scales. The focus lies on Quality planning (one item), monitoring of patient and professional opinions (six items), monitoring of the quality system (four items) and the improvement of the quality by development of staff competencies (four items). Data from the visit have the advantage that the judgment of the external visitors is based on years of experience on hospital evaluation of performance and that they are based on factual and verified data. The judgment has been made on a five-point-Likert scale (range 0–4) from ‘No or negligible compliance’ to ‘Full compliance’. The range of the Index is based on the four scales and ranges from 0 to 16 [9].

#### CQII

The Clinical Quality Implementation Index (CQII) has been designed to measure to what extent some key quality areas are implemented across the hospital. These areas are as follows: preventing hospital infections, medication management, preventing patient falls, preventing patient ulcers, routine testing of elective surgery patients, safe surgery practices and preventing deterioration. The judgment of the external visitor is based on various sources, such as group minutes, protocol checks and compliance reports of group minutes. The visitor is asking for a responsible group with regard to the key area, formal audits or reviews and the measurement of relevant outcome indicators. The judgment has been made on a five-point-Likert scale (range 0–4) from ‘No or negligible compliance’ to ‘Full compliance’. The range of the Index is based on the seven key areas and ranges from 0 to 14. [9]

### Four department-level measures

Quality development at department level was measured by four constructs [10] based on supporting evidence from the scientific literature [14–18]: (1) assigning responsibility and specialized expertise to specific professionals which was consider to stimulate clinical leadership and supports the use of evidence-based guidelines. The mean score on these items is combined in a measure called specialized expertise and responsibility (SER). (2) Evidence-based organization of pathways (EBOP), which explores items related to whether the organization of the pathway takes into account requirements for evidence-based medicine. These items differ for each pathway, as they need different contextual factors. (3) Patient safety procedures, which include a sample of recommended items to be present in the wards (hand hygiene, medication management, safe equipment, clear instructions and adverse event reporting systems). (4) Clinical review (CR) consists of three items asking whether a department has done a clinical review recently, whether this is a multidisciplinary audit against practice guidelines and whether professionals participate and get direct feedback. Answers on items for all scales were rated on a five-point-Likert scale (range 0–4) from ‘No or negligible compliance’ to ‘Full compliance’. The scale score was based on the mean score of the items of a specific scale.

## Statistical analysis

We calculated descriptive statistics for the sample of in-depth hospitals, for each of the variables used in this study. We used linear regression models to investigate (i) the relationships between hospital-level quality measures (QMSI, QMCI and CQII) and (ii) the pathway-specific relationship between each of the pathway-level quality measures (SER, EBOP, PSS and CR) and the hospital-level quality measures. Variable selection for our statistical models was guided by the use of a directed acyclic graph (DAG) in Fig. 1. DAGs are path diagrams that depict causal relationships between variables in a conceptual model, and they impart a basic set of rules that can be used to guide variable selection for a statistical model [19]. While this particular analysis was not causal in nature, we invoked the DAG to provide a simple visual aid to elucidate our covariate selection. As a result of all the hypothesized interrelationships between the variables in Fig. 1, some variables may act as confounders, or as intermediates, depending on the associational pathway of interest. For example, when we investigate the relationship between QMSI and the pathway-level variables, we did not control for QMCI or CQII as they are hypothesized to be intermediate variables. However, for the association between QMCI and the departmental-level variables, we control for QMSI because it is a predictor for both and thus represents an open backdoor path.

Associations and intraclass correlation coefficients were estimated using multivariable adjusted linear mixed models with a random intercept by country to account for clustering of hospitals within countries. All models were adjusted for fixed effects at the hospital level (ownership, hospital size and teaching status). In the first set of models designed to asses interrelationships between the hospital-level quality measures, we additionally adjusted for QMSI in estimating the association between QMCI and CQII. In the second set of models designed to assess vertical relationships between the hospital-level measures and the pathway-level measures, we additionally adjusted for QMSI and QMCI when testing the independent variables QMCI and CQII, respectively. We used SAS version 9.3 (SAS Institute, Cary, NC, USA) to conduct all analyses.

## Results

Characteristics of hospitals (*N* = 71) and departments (*N* = 283) of the study are summarized in Table 1. Although we have audit data on 74 hospitals, 3 hospitals are missing QMSI. Since QMSI is included in every model (either as the main exposure or as a confounder), we restricted the analysis dataset to hospitals that were not missing QMSI. This dropped the total number of hospitals in the analysis to 71. In every hospital, except for one with no delivery department, four departments related to the four tracer conditions participated. Most hospitals were public hospitals with 501–1000 hospital beds.

**Table 1:** Characteristics of hospitals and departments participating in the analysis

Characteristic	*N*	(%)
Number of hospitals	71	(100)
Czech Republic	12	(16.9)
France	11	(15.4)
Germany	4	(5.6)
Poland	11	(15.4)
Portugal	10	(14.0)
Spain	11	(15.4)
Turkey	12	(16.9)
Number of departments	283	(100)
AMI	71	(25.0)
Deliveries	70	(24.7)
Hip fracture	71	(25.0)
Stroke	71	(25.0)
Number of teaching hospitals	31	(43.6)
Number public hospitals	56	(78.8)
Approximate number of beds in hospital		
<200	7	(9.8)
200–500	21	(29.5)
501–1000	30	(42.2)
>1000	13	(18.3)

In Table 2, we provide the descriptive statistics for hospital- and departmental-level quality index and score variables. The average score of all departments on QMSI is 19 of 27 (SD 4.1); for QMCI it is 10 of 14; and for CQII 8 of 14. If we standardize the scores on the hospital-level measures, the score on QMSI is higher than the score on QMCI, and the QMCI score is higher than the score on CQII. All departments are part of the same sample of hospitals; therefore, the scores of the various department types are similar. For the departmental QM measures, SER and PSS departments score on average between 2 and 3 points (range 0–4). The scores are higher for EBOP and lower for CR. Comparing the four conditions, results showed lower scores for hip fracture pathways.

**Table 2:** Descriptive statistics for hospital- and departmental-level quality variables

	All departments		AMI		Deliveries		Hip fracture		Stroke	
	Mean	(SD)	Mean	(SD)	Mean	(SD)	Mean	(SD)	Mean	(SD)
Index and score variables (level) (scale range)										
Quality Management System Index (hospital level) (0–27)	19.4	(4.1)	19.4	(4.1)	19.3	(4.1)	19.4	(4.1)	19.4	(4.1)
Quality Management Compliance Index (hospital level) (0–16)	10.4	(3.0)	10.4	(3.1)	10.3	(3.1)	10.4	(3.1)	10.4	(3.1)
Clinical Quality Implementation Index (hospital level) (0–14)	8.4	(2.9)	8.4	(2.9)	8.4	(2.9)	8.4	(2.9)	8.4	(2.9)
Specialized Expertise and Responsibility (department level) (0–4)	2.6	(1.1)	2.7	(1.1)	2.8	(1.1)	2.2	(0.9)	2.7	(1.2)
Evidence-Based Organization of Departments (department level) (0–4)	3.0	(1.0)	3.2	(0.9)	3.7	(0.3)	2.3	(1.1)	3.0	(1.0)
Patient Safety Strategies (department level) (0–4)	2.6	(0.6)	2.6	(0.6)	2.7	(0.6)	2.5	(0.6)	2.5	(0.6)
Clinical Review (department level) (0–4)	1.9	(1.4)	2.1	(1.4)	2.4	(1.4)	1.4	(1.3)	1.9	(1.5)

Associational analysis between the three hospital-level QM measures showed significant positive associations between all three measures. This means that hospitals with a higher score for example on QMSI also have a higher score on QMCI and CQII. The intraclass correlation coefficients, which indicate the % variation that is attributable to between-country heterogeneity, ranged from 18 to 27% (Table 3).

**Table 3:** Associations between hospital-level QM measures (*N* = 71)

Quality index	Quality Management Compliance Index (QMCI)				Clinical Quality Implementation Index (CQII)			
	*B*	SE	pr >|t|	ICC	*b*	SE	pr >|t|	ICC
QMSI^a^ (Index 0–27)	0.417	0.082	<.001	0.213	0.303	0.081	0.001	0.271
QMCI^a,b^ (Index 0–16)					0.476	0.109	<.001	0.176

^a^Multivariate mixed linear regression with random intercept by country, and adjusted for fixed effects at the hospital level (ownership, teaching status and number of bed).

^b^Additionally adjusted for fixed effect at the hospital level: QMSI.

In Table 4, we provide results of associational analysis of QMSI, QMCI and CQII on SER, EBOP, PSS and CR for AMI, delivery, hip fracture and stroke care pathways. Results indicated a significant positive relation between QMCI for all departments with SER, the quality measure focusing on specialized expertise and assigned responsibilities, EBOP, the Evidence-based organization of care, and PSS, the patient safety strategies. The results also indicated a positive relation between QMSI for all departments with PSS and between CQII with PSS for AMI and Stroke. No associations were found between hospital-level quality measures and CR, clinical review, at AMI departments. In general, the beta coefficients were small for all investigated relationships. Intraclass correlation coefficients were substantial, ranging from 10% to >50% for all departmental quality measures except PSS. For PSS, between-country variation was negligible.

**Table 4:** Associations of QMSI, QMCI and CQII with department-level quality measure**s** (significant results of *P* < 0.05 are marked in bold)

Pathway	Hospital-level quality measure	Specialized expertise and responsibility (0–4)				Evidence-based organization (0–4)				Patient safety strategies (0–4)				Clinical review (0–4)			
		*b*	SE	pr >|t|	ICC	*b*	SE	pr >|t|	ICC	*b*	SE	pr >|t|	ICC	*b*	SE	pr >|t|	ICC
AMI (*N* = 69)	QMSI^a^ (Index 0–27)	0.026	0.030	0.383	0.511	−0.039	0.029	0.190	0.197	0.044	0.017	0.014	0.015	0.058	0.040	0.150	0.350
	QMCI^a,b^ (Index 0–16)	0.114	0.043	0.010	0.456	0.148	0.041	0.001	0.104	0.089	0.024	0.001	0.000	0.074	0.060	0.217	0.319
	CQII^a,b,c^ (Score 0–14)	0.047	0.050	0.352	0.447	0.027	0.047	0.566	0.101	0.058	0.028	0.045	0.000	0.044	0.069	0.526	0.319
Deliveries (*N* = 69)	QMSI^a^ (Index 0–27)	0.031	0.032	0.342	0.316	−0.003	0.010	0.755	0.389	0.035	0.020	0.079	0.150	0.063	0.042	0.137	0.326
	QMCI^a,b^ (Index 0–16)	0.149	0.046	0.002	0.188	0.029	0.015	0.061	0.397	0.109	0.026	0.001	0.000	0.130	0.062	0.040	0.258
	CQII^a,b,c^ (Score 0–14)	0.044	0.055	0.426	0.150	0.009	0.018	0.632	0.400	0.038	0.031	0.234	0.012	0.067	0.073	0.366	0.260
Hip fracture (*N* = 71)	QMSI^a^ (Index 0–27)	0.014	0.029	0.644	0.266	−0.045	0.027	0.010	0.574	0.043	0.017	0.015	0.000	0.080	0.036	0.030	0.342
	QMCI^a,b^ (Index 0–16)	0.176	0.039	<.001	0.148	0.113	0.039	0.005	0.578	0.089	0.025	0.001	0.000	0.125	0.053	0.021	0.405
	CQII^a,b,c^ (Score 0–14)	0.003	0.046	0.952	0.148	0.024	0.045	0.601	0.563	0.039	0.029	0.178	0.000	0.003	0.061	0.962	0.402
Stroke (*N* = 71)	QMSI^a^ (Index 0–27)	0.047	0.034	0.171	0.519	0.031	0.028	0.273	0.403	0.050	0.017	0.006	0.000	0.128	0.041	0.003	0.441
	QMCI^a,b^ (Index 0–16)	0.119	0.049	0.019	0.477	0.104	0.041	0.015	0.320	0.097	0.025	0.001	0.000	0.151	0.061	0.016	0.417
	CQII^a,b,c^ (Score 0–14)	−0.028	0.057	0.628	0.468	0.001	0.048	0.981	0.318	0.074	0.028	0.010	0.010	0.016	0.070	0.822	0.417

^a^Multivariate mixed linear regression with random intercept by country, and adjusted for fixed effects at the hospital level (ownership, teaching status and number of bed).

^b^Additionally adjusted for fixed effect at the hospital level: QMSI.

^c^Additionally adjusted for fixed effect at the hospital level: QMCI.

## Discussion

We assumed that the development of a good infrastructure of a QMS influences the compliance with QM activities and the implementation of clinical QM activities at hospital level. Our results confirmed the relationships between QMSI, QMCI and CQII. Furthermore, we assumed a relationship between QM measures at hospital level with QM activities at pathway level. The results showed a mixed picture between the influence of the three QM measures at hospitals level and the department-level QM measures. Most associations were found between QMCI and department-level measures. The lowest number of associations was found between CQII and department-level measures. There were small differences between the four types of care pathways. The relatively low beta coefficients in all investigated relationships indicated rather weak associations between QM at both levels. Between-country variability was substantial in most of our models; however, very low intraclass correlation coefficients consistently estimated for the relationship between hospital-level quality measures and the PSS measure for each pathway may be evidence of more uniform implementation of safety strategies across our sample of countries.

### Relation with earlier research

In this study, we tried to capture the QM activities in the whole hospital by using several instruments and QM measures at different levels. We showed that our QM measures were related to each other but were also sufficiently distinct to add to an overall concept of QM in multi-level complex healthcare organizations. Quality assessment of entire hospitals is mainly done during accreditation and certification processes. The impact of these forms of external assessment has been described in various articles [20–23].

External assessment by trained auditors is an intensive time-consuming and costly process. With the instruments used in our research, it is possible for hospitals to assess the maturity of their own QM systems and strategies without taking directly the step for official accreditation or certification efforts and costs. Depending on the evaluator, external assessment provides a very comprehensive snapshot of the quality maturity status, whereas the DUQuE measures presented here allow a certain level of self-assessment and diagnosis of the organization. It is a reflective rather than a prescriptive assessment and requires discussions internally. These discussion may be a stronger lever for improvement than a certification every 3 years. Thorough self-assessment is also a useful step to get insight in strength and weaknesses of one or more of the specific QM indices and scales.

It might also be possible to use the DUQuE instruments together with health insurers, healthcare inspectorates or board of trustees to assess the maturity of existing QM systems throughout the hospital.

### Strength and limitations

The DUQUE project is a cross-sectional study and does not measure improvement in itself, but the extent of implementation of QM at various levels in hospitals. Hospitals with a more developed (more mature) QM system are expected to engage more actively, in quality improvement activities at department level, which in turn are expected to improve quality for the patient expressed in better clinical outcomes. This study does not measure the relation with clinical outcomes.

While Questionnaire data reflect individuals' assessment on the availability and implementation of QM structures, activities and procedures, the QMCI and CQII from the audit are based on observation of compliance by external visitors. This mixed-method design gives more reliable results than a single-method design.

## Conclusion

Associations were found between the various QM measures, whereby hospital-level measures are positively associated with pathway-level measures. The strength of the association varies between measures and conditions. By using the seven measures of QM, it is possible to obtain a more comprehensive picture of the maturity of QM in hospitals, with regard to the different levels and across various types of hospital departments. These measures facilitate self-diagnosis of hospitals on their continuing journey towards quality improvement.

## Funding

Funding to pay the Open Access publication charges for this article was provided by European Community's Seventh Framework Programme (FP7/2007–2013) under grant agreement no. 241822.

## Appendix

The DUQuE Project Consortium comprises: Klazinga N, Kringos DS, Lombarts K and Plochg T (Academic Medical Centre-AMC, THE NETHERLANDS); Lopez MA, Secanell M, Sunol R and Vallejo P (Avedis Donabedian University Institute-Universitat Autónoma de Barcelona FAD. Red de investigación en servicios de salud en enfermedades crónicas REDISSEC, SPAIN); Bartels P and Kristensen S (Central Denmark Region & Center for Healthcare Improvements, Aalborg University, DENMARK); Michel P and Saillour-Glenisson F (Comité de la Coordination de l'Evaluation Clinique et de la Qualité en Aquitaine, FRANCE); Vlcek F (Czech Accreditation Committee, CZECH REPUBLIC); Car M, Jones S and Klaus E (Dr Foster Intelligence-DFI, UK); Garel P and Hanslik K (European Hospital and Healthcare Federation-HOPE, BELGIUM); Saluvan M (Hacettepe University, TURKEY); Bruneau C and Depaigne-Loth A (Haute Autorité de la Santé-HAS, FRANCE); Shaw C (Independent Consultant, UK); Hammer A, Ommen O and Pfaff H (Institute for Medical Sociology, Health Services Research and Rehabilitation Science, University of Cologne-IMVR, GERMANY); Groene O (London School of Hygiene and Tropical Medicine, UK); Botje D and Wagner C (The Netherlands Institute for Health Services Research-NIVEL, the NETHERLANDS); Kutaj-Wasikowska H and Kutryba B (Polish Society for Quality Promotion in Health Care-TPJ, POLAND); Escoval A (Portuguese Association for Hospital Development-APDH, PORTUGAL) and Franca M (Portuguese Society for Quality in Health Care-SPQS, PORTUGAL); Almeman F, Kus H and Ozturk K (Turkish Society for Quality Management in Healthcare-SKID, TURKEY); Mannion R (University of Birmingham, UK); Arah OA, Chow A, DerSarkissian M, Thompson C and Wang A (University of California, Los Angeles-UCLA, USA); Thompson A (University of Edinburgh, UK).
